# Status of Pediatric Cardiac Care in Developing Countries

**DOI:** 10.3390/children6020034

**Published:** 2019-02-25

**Authors:** Anita Saxena

**Affiliations:** Department of Cardiology, All India Institute of Medical Sciences, New Delhi 110 029, India; anitasaxena@hotmail.com

**Keywords:** congenital heart disease, rheumatic heart disease, developing countries

## Abstract

About 1.35 million babies are born with congenital heart disease each year globally. Most of these are expected to lead normal, productive lives if they are treated in time. However, 90% of babies born with congenital heart disease live in regions where medical care is inadequate or unavailable. The privilege of early diagnosis and timely intervention is restricted to only those born in developed countries. Added to the burden of congenital heart disease is rheumatic heart disease, which remains a global health problem in many low-income and middle-income countries. Providing optimal care for all these children is a daunting task, and requires funds and proper planning at various levels of the health care system. This article describes the burden of pediatric heart disease, including lacunae in the current state, as well as challenges and opportunities for providing optimal care to this large population of children.

## 1. Introduction

Heart diseases in children include those present at birth (congenital heart diseases) and those acquired later in life (rheumatic heart disease, cardiomyopathies, pericardial diseases, and others). Congenital heart diseases (CHD) are the most common birth defects; they are responsible for nearly one-third of all congenital birth defects [1]. Advances in pediatric cardiology and cardiac surgery have made it possible to repair or palliate most of the CHD, including the complex ones. Newborns survival rates depend on where in the world the baby is born. If access to screening, early diagnosis, and treatment is available, the baby has a 95% chance of survival to adult life and good long-term outcomes [2]. Such advanced care is practically unavailable to over 90% of babies born in developing countries, including India [3]. In addition to CHD, a large number of children suffer from rheumatic heart disease (RHD) in the very same regions where facilities for diagnosis and treatment are sparse or unavailable. 

While one cardiac center caters to a population of approximately 120,000 in North America, in Asia, a population of 16 million people is served by one center [4]. In Africa, this ratio is one center per 33 million people [4]. Similarly, the number of cardiac surgeons per person is considerably better in North America and Europe (one cardiac surgeon per 3.5 million people) as compared to Asia (one cardiac surgeon per 25 million) [5]. These figures indicate that thousands of children in Asia and Africa have none or minimal access to health care, resulting in much higher mortality compared to children born in rest of the world. Further, children with heart disease living in low-income countries tend to present late and frequently suffer from poor nutrition, infections, and pulmonary hypertension, which present further challenges to their management in resource-limited settings. Overall, the treatment of these children is a daunting task, since pediatric cardiac care is highly resource intensive, both in terms of trained staff as well as equipment and infrastructure.

## 2. Burden of the Disease in Developing Countries

### 2.1. Congenital Heart Disease

The birth prevalence of CHD is reported to be between eight and 12 per 1000 live births [5,6]. Reported figures vary worldwide, with the highest prevalence in Asia (9.3/1000 live births) and the lowest in Africa (1.9/1000 live births) [7]. The low prevalence in Africa is likely to be due to a paucity of data, lack of access to health care, lack of trained health personnel, and very early mortality due to CHD. In fact, the birth prevalence of CHD in some of the low-income countries may be higher due to increased rates of maternal infections such as rubella, and higher rates of exposure to other teratogens. Birth prevalence is also related to altitude, and may be increased by up to fivefold in high altitude regions such as Machu Pichu, Peru, etc. Most studies have shown almost uniform prevalence rates for CHD across the globe, including one from India [8]. 

Considering a rate of nine per 1000 births, about 1.35 million babies are born with CHD each year globally [9]. Approximately 240,000 children are born with CHD in India itself, while another 150,000 are born in China and only 32,000 are born in the United States (USA). Worldwide, 90% of newborns with CHD live where medical care is inadequate or unavailable [3]. CHD constitutes a major cause of infant mortality across the globe [10]. 

### 2.2. Rheumatic Heart Disease

RHD remains a global health problem in low-income and middle-income countries. The recent Global Burden of Disease study has estimated that worldwide, 33 million cases have RHD [11]. Rheumatic fever continues in high prevalence regions, and as many as 282,000 new cases are reported annually [12]. Most of the new cases are likely to be in young children aged between five and 15 years. Annually, 233,000 patients die due to rheumatic fever and RHD, which is primarily due to late diagnosis and the limited availability of cardiac surgery in these regions [12]. In addition to clinical RHD, the echocardiographic screening studies in school children have shown over 10 times more prevalence than clinical auscultation [13,14,15,16]. The disability-adjusted life year burden was estimated at 1430 days (range 944–2067) in 2010, which is approximately one quarter of the global DALY (disability-adjusted life year) burden of cancer [17].

## 3. Contributory Factors for Current Status and How to Address These Factors

Comprehensive pediatric heart care is very resource-intensive, and requires a sophisticated infrastructure. It is challenging to develop a successful and effective program in a resource-constrained environment. The first step needs to be recognition of the contributory factors, as these may vary from region to region. 

### 3.1. Delay in Diagnosis and Referral

Most CHD are not diagnosed in the antenatal period, despite the mother having undergone more than one ultrasound examination. A significant proportion of births occur at home and the infants with ductus-dependent CHD may succumb before any medical assistance can be given. For those born in hospital, a pre-discharge screening of newborns by pulse oximetry is very infrequently practiced, especially in rural and semi-urban centers. Significant delay further occurs between diagnosis and referral to a cardiac center [10]. This stems from limited knowledge about available treatment facilities for heart disease in children amongst the primary caregivers (physicians/pediatricians). Many health professionals are unaware of the advances made in the treatment of CHD and RHD, and parents are counseled against interventions due to apprehension of poor long-term outcomes. Most health staff, including physicians, nurses, and other health workers, are not sensitized to the problem of heart disease in children, and lack training in recognizing these. The general public is unaware of the significance of treating a sore throat and that an untreated sore throat can result in RHD. Often, medical assistance is not sought for sore throats. Patients with RHD often present at an advanced stage of valvular damage, when they are grossly symptomatic. Delay in referral results in suboptimal outcomes such as complications and co-morbidities (such as undernutrition, heart failure, and pulmonary hypertension) may have already set in. 

The community as well as front-line health workers need to be sensitized to the problem of heart disease in children. Electronic and print media should be utilized for this purpose. The pediatricians and other health workers should be educated about CHD and RHD as well as informed about the importance of early diagnosis and treatment. They should be informed about the advancements that have occurred for children with heart diseases. For RHD patients, the health staff can play a very important role in emphasizing the need for secondary prophylaxis and ensuring long-term compliance.

### 3.2. Shortage of Pediatric Cardiac Care Centers and Trained Personnel

Another important contributing factor is the severe shortage of pediatric cardiac centers and low numbers of trained work force in developing countries. One cardiac center caters to a population of 120,000 in North America. In Asia, the ratio is one to 16 million, and in Africa, the ratio is one to 33 million [4]. According to a recent study, levels of cardiac surgery range from 0.5 per million in the low-income and lower–middle-income countries (average 107 ± 113 per million; representing a population of 1.6 billion) to 500 in the upper–middle-income countries (average 270 ± 163 per million; representing a population of 1.9 billion). The data shows that a significant degree of under delivery of lifesaving open heart surgery prevails in low-income countries [18].

The small number of pediatric cardiac facilities are usually clustered in large metropolitan cities, and are thus inaccessible to the large percentage of the population living in rural areas far away from the centers. Families of affected children have to travel thousands of kilometers to bring their children for surgery and further follow-up visits. In most instances, it becomes financially nonviable. In one study from South America, for each 100 km of distance traveled, patients were 1.4 times more likely to be above the median age for their surgical class at the time of surgery (odds ratio (OR) 1.4; 95% confidence interval (CI) 1.04, 1.9; *p* = 0.002) [19]. In their study, there was a significant difference when rural versus urban residence was considered. Children from rural areas were almost three times more likely to be above the median age for their surgical class at the time of surgery (*p* = 0.001). Meeting these challenges requires knowledge of local settings. According to a publication in 2007, an estimated 15 million children die or are crippled annually by potentially treatable or preventable cardiac disease [20].

Specific measures include establishing more centers that can cater to the needs of neonates, infants, and children with CHD. Developing a sustainable and comprehensive pediatric cardiac center is a challenging task because of the large investments required in terms of sophisticated technology, infrastructure, and trained personnel. The center needs a trained motivated team of health professionals, including cardiologists, surgeons, intensivists, and other cardiovascular specialists. For large countries, it is important that these centers are geographically distributed, so that families do not need to travel long distances where the local environment and language is different. At the same time, each center must have a minimum number of cases to maintain the skills of the trained staff. In-country, structured training programs (as in Brazil) for cardiologists, surgeons, intensivists, and anesthesiologists are necessary for the sustainability and growth of this subspecialty. China has developed in-country training programs that have been shown to be feasible and cost-effective [21], and more countries are following with the same. In addition, the Internet and telemedicine are being successfully used to transfer knowledge and skills [22]. 

### 3.3. Financial Constraints

Most low-income countries spend a very small proportion of their gross domestic product on health care. The allocated budget is often used for meeting the basic health needs such as infections, malnutrition, etc. The pediatric heart diseases usually take a back seat, and are not a priority. Medical insurance is practically nonexistent in most developing countries. In most instances, families are expected to pay for the treatment out of their pocket, which they can barely afford. In a study from Kerala, India, surgery for CHD resulted in significant financial burden for the majority of families [23]. Approximately half of the families borrowed money during the follow-up period after surgery. According to a recently published report from India, about 35% of cardiac surgeries are funded by families themselves [24]. Government schemes cover about 40% of all cardiac surgeries for CHD patients. Many hospitals partner with charitable non-governmental organizations and multinational companies to assist economically weaker families. About 20% of cardiac surgeries are funded by such organizations [24]. Developing partnerships through non-governmental organizations and governments has shown to be helpful in capacity building to make pediatric cardiac program sustainable. It is estimated that 58% of the burden of CHD can be averted by scaling up selected surgical care in low-income and middle-income countries through various means [25]. 

Transporting children with CHD or RHD to centers in affluent countries for surgery is not only very expensive, it is also a non-sustainable model. Similarly, the visit of an expert operating team to developing countries can also work for a short time, but it has the advantage of capacity building the home center. The pediatric cardiac unit in Guatemala city is an excellent example of a program that was established through a collaboration between the Aldo Castaneda Foundation and the government of Guatemala. 

Several other methods of cost containment have been described. Providing care for both children and adults under one roof is one such example where equipment and manpower are shared. However, such centers may not have expertise in treating neonates and infants. Encouraging homegrown technology, and exclusion of the “middle man” for importing equipment and consumables significantly cuts down the cost. Incorporating research into a program is very important and helps with its sustainability. 

### 3.4. Inadequate Preventive Measures and Screening

So far, little emphasis has been placed on preventive measures for CHD or RHD. This needs to be stressed, as the investment that is required is much smaller. Mass immunization against rubella should be the starting point at the national level. Similarly, the early diagnosis and timely treatment of a bacterial sore throat can help prevent rheumatic fever. Although one can have a specific preventive program for children with CHD and RHD, a more comprehensive program that caters to the well-being of children in general, and incorporates a number of other common disorders is more likely to be sustainable. Screening newborns with pulse oximetry to diagnose critical CHD should become a part of newborn care.

## 4. Conclusions

Most children with heart diseases (CHD or RHD) living in developing countries do not have access to quality care. It is well established that the treatment of CHD and RHD is cost-effective, and this justifies the concept of pediatric cardiac care. However, a number of challenges/barriers need to be overcome before an average child living in a developing country has access to comprehensive cardiac care. Over the last decade or so, some of these challenges have been addressed. Several new centers have been established, trained manpower is increasing, and newer cost-effective strategies and innovations are being developed. As the burden of these patients is huge, a lot more needs to be done before a real impact on outcome becomes obvious for children with heart diseases. Early diagnosis requires sensitization of the community and health workers for pediatric heart diseases. This may be the easy part, however; managing children that are diagnosed with CHD or RHD requires significant resources and infrastructure. The resources are not only limited, but sometimes suboptimally utilized. The challenges of providing optimal care for an average child with heart disease are unparalleled, and will lead to innovations and opportunities. In addition, a collective effort by the government, non-governmental organizations, physicians, and other caregivers will be required for continued improvement in health care delivery. One remains optimistic for future growth and progress. All efforts to improve the care of children with heart disease must be complemented with research that is locally relevant.
